# Orphan Drug Approval in Canada, 1999-2022: A Cross-sectional Study

**DOI:** 10.34172/ijhpm.8916

**Published:** 2025-09-28

**Authors:** Joel Lexchin

**Affiliations:** ^1^School of Health Policy and Management, York University, Toronto, ON, Canada.; ^2^Department of Family and Community Medicine, University of Toronto, Toronto, ON, Canada.

**Keywords:** Orphan Drugs, Health Canada, Food and Drug Administration, Drug Approval, Therapeutic Value, Therapeutic Class

## Abstract

The number of drugs for orphan indications has been increasing significantly in Canada and the federal government recently announced an investment of $1.5 billion dollars over 3 years primarily directed at helping to fund the cost of these drugs. There are claims and counterclaims about what percent of Food and Drug Administration (FDA) orphan drugs are available in Canada and how delayed these drugs are in being approved by Health Canada. This study uses FDA and Health Canada databases and data from three health technology assessment agencies and one drug bulletin to provide objective data about the percent of FDA approved drugs that were also approved by Health Canada, any delays in Canadian approval and the additional therapeutic value of new orphan drugs. Decisions about what drugs should be publicly covered and how long it took to make those decisions were not investigated. From 1999 to 2022, the FDA approved 326 new drugs for an orphan indication and Health Canada approved 231 (70.9%) for the same indication. The median time between FDA and Health Canada approval was 346 days (interquartile range [IQR] 181, 785). The percent rated as major improvements declined from 50% of the total in 2004-2008 to 13.6% in 2019-2022. These findings need to be taken into account as Canada develops an orphan drug policy and decides on criteria for funding this group of drugs. Specifically, when high quality evidence about the additional therapeutic value of orphan drugs is not available at the time of approval, risk sharing funding agreements with manufacturers should be put in place. Manufacturers should understand that if the results of post-market trials do not provide convincing evidence of value, funding will be withdrawn. Finally, the quality of any research plan should be used to prioritize candidates for federal funding.

## Introduction

 The number of orphan drugs approved annually by Health Canada has increased significantly from 36% of the total in 2012-2015 to 51% in 2019-2022.^1^ In March 2023 Health Canada announced that the federal government would be investing $1.5 billion over three years in support of a National Strategy for Drugs for Rare Diseases with $1.4 billion going to the provinces and territories to help improve access to new and emerging drugs. Additionally, the government would be providing $33 million to Indigenous Services Canada’s Non-Insured Health Benefits Program to support people with orphan diseases and $68 million for various other initiatives related to orphan diseases.^2^

 However, public funding to improve access to orphan drugs is only relevant if the drugs are approved in the first place. On its website, the Canadian Organization for Rare Disorders claims, without providing any substantiating evidence, that “Only 60% of treatments for rare disorders make it into Canada and most get approved up to six years later than in the USA.”^3^ Rawson and Adams in their analysis of health technology assessment, price negotiation, and government formulary listing for orphan drugs state that only 39% of the orphan drugs submitted in 2020 to the United States Food and Drug Administration (FDA) and/or the European Medicines Agency were submitted to Health Canada.^4^ However, the claims in their article have been contested.^5^

 This study looks at what percent of orphan drugs approved by the FDA between 1999 and 2022, inclusive, were also approved by Health Canada for the same indication and what the time difference was between FDA and Health Canada approval. Public funding decisions for this group of drugs were not investigated. Additionally, the additional therapeutic value and therapeutic classes of orphan drugs both approved by Health Canada and that were never submitted to or approved by Health Canada were examined.

## Methods

###  List of Orphan Drugs Approved by the FDA

 A list of all drugs (new molecular entities and biologics license applications) initially approved as orphan drugs by the FDA between 1999 and 2022 was generated from annual lists of FDA approvals.^6,7^ Drugs not initially approved for orphan indications but that subsequently received orphan status for new indications were not considered. Brand and generic names, the date of approval and the orphan indication(s) were recorded on an Excel spreadsheet for each drug. 1999 was chosen as the starting year because lists of FDA approvals in prior years were not publicly available and 2022 as the final year because the median wait time between submission of the drugs to the FDA and Health Canada is almost 12.5 months.^4^

###  List of Orphan Drugs Approved by Health Canada

 Approval by Health Canada of the same drug for the same indication and the date of approval was determined by consulting one of three sources: Summary Basis of Decision documents,^8^ the Product Monograph^9^ and the Notice of Compliance database.^10^ In addition, the websites for drug applications still under consideration and those that had been rejected or where applications were withdrawn by the sponsor were searched.^11^ The date of Health Canada approval was recorded along with the names of drugs still being reviewed, rejected or withdrawn.

###  Therapeutic Indications for Drugs

 The World Health Organization (WHO) Anatomical Therapeutic Chemical/Defined Daily Dose Index was used to categorize the drugs at the second level.^12^

###  Additional Therapeutic Value of New Drugs

 Therapeutic value was determined by searching the databases of four organizations that evaluate the efficacy and safety of new drugs: Annual reports of the Patented Medicine Prices Review Board, the Canadian agency that evaluates whether the price of patented drugs is excessive; Prescrire International, an independent French drug bulletin; the Haute Autorité de Santé, the French body that assesses the efficacy and safety of drugs; and the Institute for Quality and Efficiency in Health Care, the German health technology assessment organization.^13-16^ All four organizations report in English and use ordinal ratings that allow discrete categorization of the additional therapeutic value. Their assessments are rigorous and systematic, and their ratings have been used by other researchers^17^ (See Supplementary file 1 for an explanation of the methodology used by each of the four organizations). Ratings were categorized as major, moderate and minor. If more than one organization assessed a drug, the highest rating was used.

###  Data Analysis

 FDA approval dates were divided into 5 time periods: 1999-2003, 2004-2008, 2009-2013, 2014-2018, 2019-2022, in order to explore trends in both the percent of FDA approved orphan drugs also approved by Health Canada and changes in additional therapeutic value of the approved drugs. The number of FDA approvals in each time period as well as the number and percent approved by Health Canada were counted. The median time (interquartile range, IQR) between FDA and Health Canada approval was calculated in days and the time differences were compared across the 5 time periods.

 The dates of Health Canada approvals were also combined into the same 5 time periods and the additional therapeutic value of the drugs approved in each period was compared. The number of drugs with major, moderate or minor additional therapeutic value either not submitted to Health Canada or not approved over the entire time period 1999-2022 was counted as were the therapeutic classes of this group of drugs.

 Kruskal-Wallis and chi-square tests were used as appropriate using Prism 10.4.0 (GraphPad Software, LLC) with a *P* value of <.05 being statistically significant.

 All data were collected by a single individual from October 10 to November 7, 2024.

## Results

 The FDA approved 326 new molecular entities or biologics license applications between 1999 and 2022 inclusive, ranging from 24 in 1999-2003 to 106 in 2014-2018. Health Canada approved 231 (70.9%) of these drugs for the same indication, ranging from 83.3% in the 1999-2003 period to 60.0% between 2019-2022. The difference in percent approved by Health Canada over the 5 time periods was statistically significantly different, *P* = .032 (Chi-square test). The lower percent in the latest period is due to only 28 (57.1%) out of 49 FDA approved drugs in 2021 and 2022 being approved by Health Canada for an orphan indication, with the remaining 22 either not submitted to Health Canada or not approved as of November 7, 2024. Of the 96 drugs not approved by Health Canada for an orphan disease over the entire time period 1999-2022, Health Canada was currently reviewing 3 drugs approved by the FDA between 2019-2022; the sponsor had withdrawn the application for 3 drugs (approved by the FDA between 2014-2018) and Health Canada had approved 6 drugs for non-orphan conditions (1 each in 1999-2003 and 2014-2018, 2 in 2009-2013 and 2 in 2019-2022) (Table 1 and Supplementary file 2). Out of 27 orphan drug submissions to the FDA in 2020, 13 had been submitted to Health Canada and approved and 1 was under review as of the end of 2024. Therefore 51.9% had been submitted as opposed to the 39% figure cited by Rawson and Adams.

**Table 1:** Drugs Approved by the Food and Drug Administration and by Health Canada for Orphan Indications, 1999-2022

	**1999-2022**	**1999-2003**	**2004-2008**	**2009-2013**	**2014-2018 **	**2019-2022**
Number of orphan drugs approved by FDA	326	24	36	55	106	105
Number (%) of same drugs for same indications approved by Health Canada^a^	231 (70.9)	20 (83.3)	29 (80.6)	39 (70.9)	80 (75.5)	63 (60.0)
Time difference (days) between Health Canada and FDA approval (median, IQR)^b^	346 (181, 785)	259 (64, 1024)	694 (213, 1470)	346 (119, 987)	322 (192, 801)	346 (194, 546)
Number of drugs being considered by Health Canada as of October 25, 2024	3	0	0	0	0	3
Number of drugs with applications withdrawn by sponsor as of October 25, 2024	4	0	0	0	3	1
Number of drugs approved by Health Canada but not for an orphan indication	6	1	0	2	1	2

Abbreviations: FDA, Food and Drug Administration; IQR, interquartile range. ^a^ Statistically significant difference between time periods, *P* =.032 (Chi-square test). ^b^ No difference between time periods, *P* =.272 (Kruskal-Wallis test).

 The time difference between FDA and Health Canada approval for the entire period was 346 (IQR 181, 785) days, ranging from 259 (IQR 64, 1024) in 1999-2003 to 694 (IQR 213, 1470) in 2004-2008. There was no statistically significant difference in the delay between the time periods, *P* = .272 (Kruskal-Wallis test) (Table 1). There were 10 (4.3%) drugs where the FDA approved the product more than 6 years before Health Canada approved it for the same indication and 3 (1.3%) where Health Canada approved the drug for an orphan indication more than 6 years before the FDA did so (Supplementary file 3).

 Health Canada approved 213 orphan drugs between 1999 and 2022 (Table 2). (The figure of 231 drugs in Table 1 refers to how many of the 326 drugs that the FDA approved were also approved by Health Canada at any time, ie, Health Canada could have approved them before 1999 or after 2022. The figure of 213 drugs in Table 2 refers only to how many drugs Health Canada approved during the period 1999 to 2022).

**Table 2:** Additional Therapeutic Value of Orphan Drugs Approved by Health Canada, 1999-2022

	**Entire Time Period**	**1999-2003**	**2004-2008**	**2009-2013**	**2014-2018 **	**2019-2022**
Number of orphan drugs approved by Health Canada	213	9	20	38	70	76
Number (%) without a therapeutic evaluation	25 (11.8)	2 (22.2)	0 (0.0)	1 (2.6)	6 (8.6)	17 (22.4)
Number (%) with a therapeutic evaluation^a^	187 (87.8)	7 (77.8)	20 (100.0)	37 (97.4)	64 (91.4)	59 (77.6)
Minor (n, %)	80 (42.8)	4 (57.1)	6 (30.0)	11 (29.7)	24 (37.5)	35 (59.3)
Moderate (n, %)	57 (30.5)	1 (14.3)	4 (20.0)	11 (29.7)	25 (39.1)	16 (27.1)
Major (n, %)	50 (26.7)	2 (28.6)	10 (50.0)	15 (40.5)	15 (23.4)	8 (13.6)

^a^ Distribution of additional therapeutic value in different time periods statistically significantly different *P* =.0081 (Chi-Square).

 Out of those drugs 187 (87.8%) had a therapeutic evaluation. Eighty (42.8%), 57 (30.5%) and 50 (26.7%) were rated as offering minor, moderate and major, respectively, additional therapeutic gains either compared to existing treatments or based on an assessment of the new drug’s overall benefit to harm ratio with respect to the disease being treated. Over the 5 time periods there was a statistically significant difference in the distribution between the three categories of therapeutic gain, *P* = .0081 (Chi-square). The percent of drugs offering a major additional therapeutic gain reached 50.0% in the 2004-2008 period but declined to 13.6% in 2019-2022 (Table 2).

 Evaluation by one or more of the four organizations of the additional therapeutic value was available for 34 (35.1%) of the 96 FDA approved products not approved by or submitted to Health Canada for an orphan indication. (The other 62 had not been evaluated by any of the four organizations). Twenty-nine (29.9%), 2 (2.1%) and 3 (3.1%) were rated as having minor, moderate and major additional value, respectively (Table 3).

**Table 3:** Additional Therapeutic Value of Drugs not Submitted to or not Approved by Health Canada, 1999-2022

**Total number of orphan drugs not approved by Health Canada or not** **approved for an orphan indication**	**Additional Therapeutic Value**			
	**Minor (n, %)**	**Moderate (n, %)**	**Major (n, %)**	**No Evaluation (n, %)**
96	29 (29.9)	2 (2.1)	3 (3.1)	62 (64.6)

 Distribution of WHO anatomic-therapeutic-chemical categories (2nd level) is broadly similar between the orphan drugs approved by the FDA and Health Canada and drugs either not submitted to or not approved by Health Canada. Antineoplastic agents, other alimentary and metabolism products and immunosuppressants forming the three largest categories in all three cases and the distribution by categories was not statistically significantly different, *P* = .155, Kruskal-Wallis test (data not shown).

## Discussion

 Out of the 326 new drugs that the FDA approved between 1999 and 2022 for an orphan indication, Health Canada approved 231 (70.9%) for the same indication. The fall off in the approval rate in 2019-2022 could be due to a decrease in the submission rate by manufacturers or a reflection of the typical delay between submissions to the FDA and Health Canada or a combination of both factors. Future research will be needed to see if this decrease represents a trend or is just a transient change.

 The median difference between FDA and Health Canada approval was 346 days or just under 1 year in favour of the FDA. There was no evidence to back up the assertion by the Canadian Organization for Rare Disorders that most orphan drugs get approved by Health Canada more than 6 years after FDA approval. Nor is the Health Canada submission rate for 2020 as low as Rawson and Adams found. The difference between the two figures is likely due to additional drugs being approved by Health Canada after Rawson and Adams finished collecting data.

 Almost 90% of the orphan drugs approved by Health Canada had their additional therapeutic value evaluated by one or more of four organizations. More than 40% were rated as offering minor additional value while the remainder were more or less evenly split between major and moderate additional value. However, there has been a significant shift in the percent offering major additional value over the years. In the 2004-2008 period, 50% of the orphan drugs approved by Health Canada were rated as a major therapeutic improvement while by 2019-2022 that figure was down to 13.6%. This decrease in the percent rated as a major therapeutic improvement is consistent with what Kesselheim and colleagues found more generally between 1987 and 2014. They concluded that the increased prevalence in the use of FDA expedited approval programs could not be attributed to an increase in the number of innovative new drug classes over time.^18^ At the same time, designing trials for orphan drugs presents a number of challenges when small patient numbers limit power and smaller trials may make the ability to evaluate additional therapeutic benefit more difficult.^1^

 While fewer orphan drugs are approved in Canada compared to the US, the distribution by therapeutic class is the same in both countries as is the therapeutic class distribution of orphan drugs not submitted to or approved by Health Canada. With regard to this latter group of 96 drugs, therapeutic evaluations were available for 35 and 29 of these were rated as minor improvements. These statistics suggest that the orphan drugs not available in Canada are not clustered in a small number of therapeutic classes and are not therapeutically important.

 These changes in the percent of FDA approved orphan drugs that are also approved in Canada and the therapeutic value of the drugs should be taken into account in determining how the federal government allocates the $1.4 billion it has set aside for funding orphan drugs^19^ as the amount committed will not be large enough to fund most of the orphan drugs approved in Canada. In 2021, the sales value of just three orphan drugs was over $1.4 billion.^20^

###  Limitations

 All the data was gathered by a single individual and that may have introduced errors. The ability to rate the therapeutic value of orphan drugs not available in Canada is limited since almost two-thirds were not evaluated by any of the four organizations. This study only examined orphan drug approvals of new drugs. Similarities and differences between the FDA and Health Canada in approvals for new orphan indications for drugs already on the market may not be the same. This study also did not evaluate what percent of orphan drug approvals are subsequently publicly funded nor how long the process of obtaining public funding takes.

## Conclusion

 Seventy percent of new drugs with an FDA orphan drug indication is approved by Health Canada about one year after FDA approval although this percent may be decreasing. In recent years, only a minority of these drugs were rated as offering major therapeutic improvement over existing drugs.

 If future research finds that there is a substantial number of therapeutically valuable orphan drugs that are not being introduced into Canada, then Health Canada should consider mechanisms for encouraging manufacturers to market this group of drugs, perhaps by instituting a new regulatory pathway for them. Health Canada should also strongly encourage manufacturers to have postmarket trials for orphan drugs underway when submissions for approval are filed. If high quality evidence about the additional therapeutic value of orphan drugs is not available at the time of approval, Lexchin and Sirrs^19^ recommend that risk sharing funding agreements with manufacturers should be put in place. Manufacturers should be willing to participate in meaningful evidence-generation with the understanding that if the results of post-market trials do not provide convincing evidence of value, funding will be withdrawn. Finally, the quality of any research plan should be used to prioritize candidates for federal funding.

## Ethical issues

 All data were publicly available and ethics approval was not required. No patients were involved in this study.

## Conflicts of interest

 Between 2021-2024, Joel Lexchin received payments for writing a brief on the role of promotion in generating prescriptions for a legal firm, for being on a panel about pharmacare and for co-writing an article for a peer-reviewed medical journal. He is a member of the Boards of Canadian Doctors for Medicare and the Canadian Health Coalition. He receives royalties from University of Toronto Press and James Lorimer & Co. Ltd. for books he has written. He has received funding from the Canadian Institutes of Health Research in the past.

## Supplementary files


Supplementary file 1. Summary of Methodology Used by the Four Organizations for Evaluating Additional Therapeutic Value of New Drugs.

Supplementary file 2. FDA Approvals for New Drugs for an Orphan Indication, 1999-2022.

Supplementary file 3. Health Canada Approvals for New Drugs for an Orphan Indication, 1999-2022.
