# Supplementary material for: Orphan Drug Approval in Canada, 1999-2022: A Cross-sectional Study
Source: Int J Health Policy Manag. 2025 Sep 28;14:8916. doi: 10.34172/ijhpm.8916 (PMC12595579; doi:10.34172/ijhpm.8916)
Supplement: Supplementary file 1 — Summary of Methodology Used by the Four Organizations for Evaluating Additional Therapeutic Value of New Drugs. [file ijhpm-14-8916-s001.pdf]

Article title: Orphan Drug Approval in Canada, 1999-2022: A Cross-sectional Study

Journal name: International Journal of Health Policy and Management (IJHPM)

Authors' information: Joel Lexchin[1,2]*

[1]School of Health Policy and Management, York University, Toronto, ON, Canada.

[2]Department of Family and Community Medicine, University of Toronto, Toronto, ON, Canada.

***Correspondence to:** Joel Lexchin; Email: jlexchin@yorku.ca

Citation: Lexchin J. Orphan drug approval in Canada, 1999-2022: a cross-sectional study. Int J Health Policy Manag. 2025;14:8916. doi:10.34172/ijhpm.8916

Supplementary file 1. Summary of Methodology Used by the Four Organizations for Evaluating Additional Therapeutic Value of New Drugs

Haute Autorité de Santé (HAS)

The Therapeutic Committee (TC) of HAS evaluation of the clinical added value of new medicines is an assessment of the therapeutic progress in terms of efficacy or safety compared with existing alternatives. The TC pays particular attention to the following criteria:

1) the comparison and choice of comparators, the methodological quality of the study, the appropriateness of the population included for the indication, the relevance of the clinical endpoint and its significance;
2) the effect size in terms of clinical efficacy, quality of life and safety;
3) the clinical relevance of this effect compared to clinically relevant comparators in view of the medical need.

Institut für Qualität und Wirtschaftlichkeit im Gesundheitswesen (IQWiG)

IQWiG is independent of industry, insurance funds and governmental institutions. The main criterion in the assessment of an examination or treatment method is the benefit for the patients. Does the intervention prolong life or reduce symptoms? Does it improve the quality of life of the patient? To answer these questions, it regularly asks affected patients about their perspectives. IQWiG assessments are not based on personal opinions or therapeutic conventions but on evidence-based scientific evidence from the worldwide medical literature. It regularly includes medical experts in its assessments to ensure that knowledge from everyday health care is considered in its reports. In order to research its evaluations about additional therapeutic benefit it cooperates closely with other research institutions and networks on a national and international level.(1, 2)

Patented Medicine Prices Review Board (PMPRB)

The PMPRB's independent Human Drug Advisory Panel (HDAP) determines the therapeutic value of each product it reviews. In deciding on the level of therapeutic innovation HDAP considers two primary factors: increased efficacy and reduction in incidence or grade of important adverse reactions and nine secondary factors: route of administration, patient convenience, compliance improvements leading to improved therapeutic efficacy, caregiver convenience, time required to achieve the optimal therapeutic effect, duration of usual treatment course, success rate, percentage of affected population treated effectively and disability avoidance/savings. The primary factors are given the greatest weight, followed by an assessment of any additional improvement as a result of the secondary factors.(3)

Prescrire International

Prescrire assesses the therapeutic value of medicines through a multistep process. First, it examines the condition or clinical setting for which the drug is proposed; then the natural course of the disease, the efficacy and safety of existing treatments, and the most relevant outcome measures. This is followed by a systematic search for clinical data on the efficacy and adverse effects of the new drug, and an assessment of the level of evidence. Based on its independent analysis of clinical data, it forms a judgement as to whether or not the new drug is beneficial for patients or whether or not its harmful effects outweigh the benefit.(4)

References

1. IQWiG. General methods Köln; 2023 [updated September 19. 7.0:[Available from: https://www.iqwig.de/methoden/general-methods_version-7-0.pdf.

2. IQWiG. Independent, patient-orientated, evidence-based Köln; nd [Available from: https://www.iqwig.de/en/about-us/methods/.

3. Patented Medicine Prices Review Board. Compendium of policies, guidelines and procedures - reissued June 2013: PMPRB; 2014 [Available from: http://www.pmprb-cepmb.gc.ca/view.asp?ccid=492#1637.

4. Prescrire Editorial Staff. Prescrire's ratings system: gauge the usefulness of new products at a glance Paris; 2011 [Available from: http://english.prescrire.org/en/81/168/46800/0/NewsDetails.aspx.
